# First stage of** labour duration and associated risk of adverse neonatal outcomes**

**DOI:** 10.1038/s41598-023-39480-0

**Published:** 2023-08-02

**Authors:** Louise Lundborg, Katarina Åberg, Anna Sandström, Xingrong Liu, Ellen L. Tilden, Jenny Bolk, Linnea V. Ladfors, Olof Stephansson, Mia Ahlberg

**Affiliations:** 1 Clinical Epidemiology Division, Department of Medicine, Solna, Karolinska Institutet, Stockholm, Sweden; 2 Division of Obstetrics, Department of Women’s Health, Karolinska University Hospital, Stockholm, Sweden; 3 Department of Nurse-Midwifery, Oregon Health & Science University School of Nursing, Portland, OR USA; 4 Department of Obstetrics and Gynecology, Oregon Health & Science University School of Medicine, Portland, OR USA; 5 Department of Clinical Science and Education Södersjukhuset, Karolinska Institutet, Stockholm, Sweden; 6 Sachs’ Children and Youth Hospital, South General Hospital, Stockholm, Sweden

**Keywords:** Epidemiology, Pregnancy outcome

## Abstract

Prior evidence evaluating the benefits and harms of expectant labour duration during active first stage is inconclusive regarding potential consequences for the neonate. Population-based cohort study in Stockholm-Gotland region, Sweden, including 46,040 women (Robson 1), between October 1st, 2008 and June 15th, 2020. Modified Poisson regression was used for the association between active first stage of labour duration and adverse neonatal outcomes. 94.2% experienced a delivery with normal neonatal outcomes. Absolute risk for severe outcomes increased from 1.9 to 3.0%, moderate outcomes increased from 2.8 to 6.2% (> 10.1 h). Compared to the reference, (< 5.1 h; median), the adjusted relative risk (aRR) of severe neonatal outcome significantly increased beyond 10.1 h (> 90th percentile) (aRR 1.53, 95% CI 1.26, 1.87), for moderate neonatal outcome the aRR began to slowly increase beyond 5.1 h (≥ 50 percentile; aRR 1.40, 95% CI 1.24, 1.58). Mediation analysis indicate that most of the association was due to a longer active first stage of labour, 13% (severe neonatal outcomes) and 20% (moderate neonatal outcomes) of the risk was mediated (indirect effect) by longer second stage of labour duration. We report an association between increasing active first stage duration and increased risk of adverse neonatal outcomes. We did not observe a clear labour duration risk threshold.

## Introduction

Over the last decades efforts have been made to redefine normal labour duration^1–6^. Contemporary research suggests that the first stage of labour can last longer than previously understood without evidence of an increase in risks for the mother and neonates^7,8^. Strategies supporting greater patience and less interventions during the first stage of labour have been tested and adopted in some settings^3,5,9,10^, however, it is an ongoing challenge in maternity care to balance the benefits of expectantly managing longer labour durations without jeopardizing the health of the neonate^7,11,12^.

Prior evidence evaluating the benefits and harms of expectant labour duration during the active phase is inconclusive regarding potential consequences for the neonate^4,12–14^. More research has been accomplished addressing second stage duration and adverse neonatal outcomes, potentially based on the idea that second stage is the most demanding period of labour for both neonate and woman^15–17^.

Labour is a continuous process and time-based interventions during both first and second stage are influenced by overall labour duration in complex ways^18,19^. In addition, many studies dichotomize labour duration into ‘prolonged’ and ‘not prolonged’ with varying time thresholds making it difficult to estimate a possible effect of a gradually increasing labour duration^13,15,20^. Recent research of the active phase of labour proposes dilation of 5 cm as the threshold separating latent and active phase, which is the updated definition according to World Health Organization (WHO)^21^. This study aimed to investigate the possible associations between active first stage of labour and neonatal outcomes in a contemporary cohort of women.

Informed by our previous research on labour duration, including both latent, active phase and second stage, we explored associations between a wide range of labour duration measures and adverse neonatal outcomes during the spontaneous onset of labour^2,17,18,22^. The objective of this study was to (a) investigate the associations between first stage duration and adverse neonatal outcomes (total effect), and (b) quantify the extent to which these associations are mediated by second stage labour duration (indirect effect).

## Material and methods

### Data sources

We conducted a population-based cohort study, using data the Stockholm-Gotland Obstetric Cohort^23^. This prospective dataset includes 334,138 maternal and neonate dyads with singleton births between January 2008 and June 2020^23^. Standardized recorded variables and data is captured directly from the electronic medical record system, by the unique personal identify number of the mother and infant, including diagnoses according to the Swedish version of the International Classification of Diseases (ICD), Tenth Revision. Furthermore, the data contain granular information embedded in standardized partographs with serial information (i.e., the date and time points of an individual’s cervical dilation). The cohort is linked to the Swedish Neonatal Quality (SNQ) register, which collects data on all infants born in Sweden who are admitted for neonatal care, including delivery rooms deaths and ICD codes. A validation of the SNQ register from 2019 demonstrated excellent accuracy^24,25^.

### Study population

**Figure 1:**
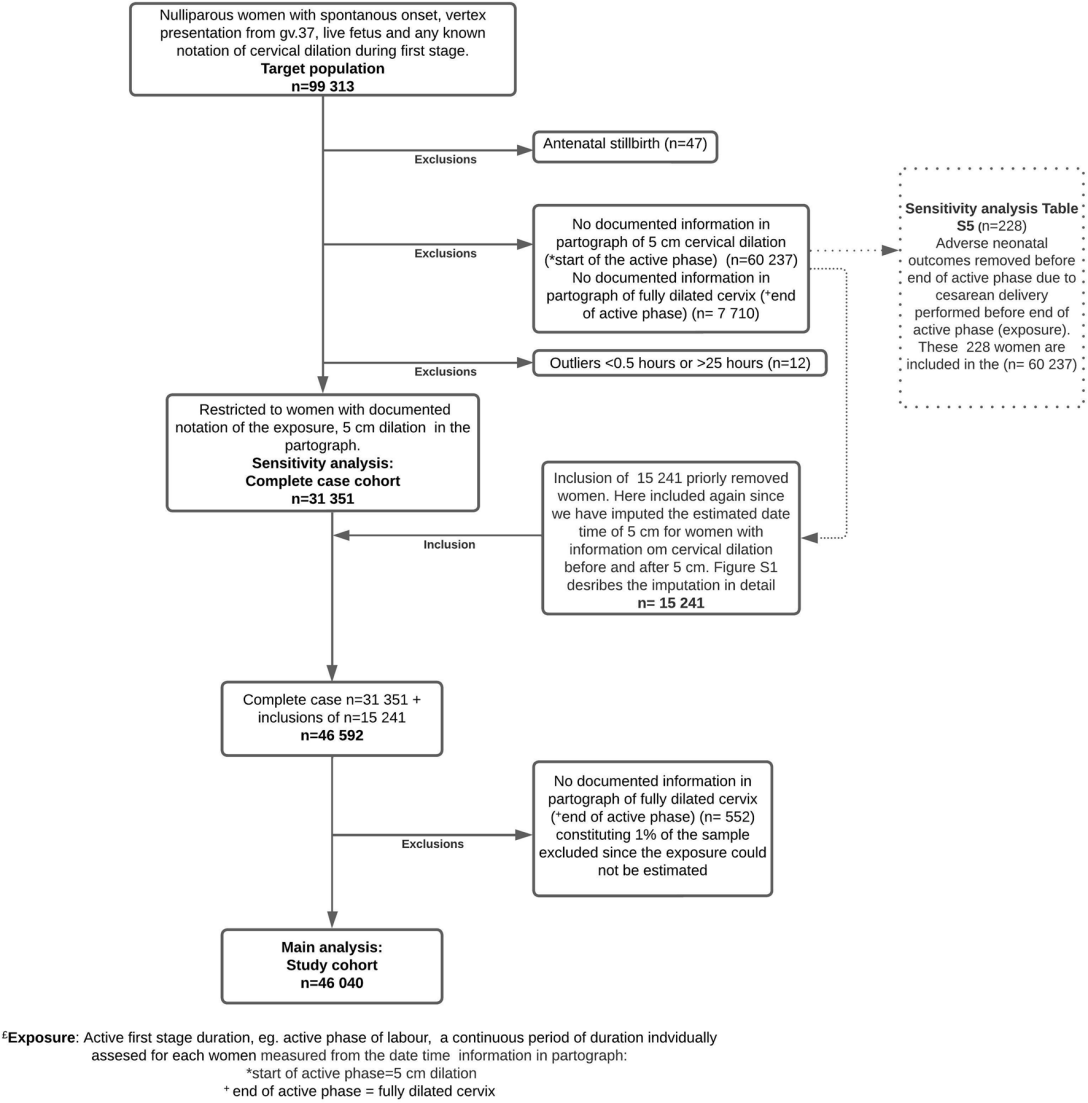
Flow chart for the target population, complete case cohort (for sensitivity analysis) and main cohort: Study Cohort N = 46,040 women.

We included nulliparous women with singleton term gestation pregnancies (≥ 37 + 0 weeks) in vertex presentation who experienced spontaneous labour onset, (n = 99,313, Target Population). Figure 1 show a flowchart for this selection of the population. From this population, we identified the “Complete case cohort”, restricted to women with a documented notation of date and time of 5 cm cervical dilation in the partograph, this cohort was used for sensitivity analysis.

For 15,241 women with missing notations for 5 cm but with measurements of cervical dilatation before and after 5 cm (see “Imputation section” for further description), we estimated and imputed the date time 5 cm and hence included these in the Study cohort which consisted of 46,040 women (Figure S1). Main analysis was conducted on the Study cohort. Data did not capture gender preferences; the standardized gendered terms are used (woman) for the individuals included in this study.

### Exposure

The exposure of interest was active phase, i.e. active first stage duration. For each included woman we identified by the first partograph notation of cervical dilation of 5 cm (known or estimated). The end of first stage was defined by the first timepoint that full cervical dilation was recorded in the partograph by the caregiver for the patient in real time. This definition of cervical dilation in relation to active phase was chosen to correspond with the updated WHO partograph and definitions^21^. Consequently, women with a caesarean delivery prior to a fully dilated cervix, during active first stage were not included.

### Imputation

The onset of active first stage (i.e. timepoint of cervical dilation of 5 cm) was not available in a large proportion of women (n = 60,237, 60%). This is to be expected given wide variability in both timing of hospital admission and of cervical examinations in labours with spontaneous onset. To improve precision and minimize the risk of selection bias, 10 different imputation patterns were hierarchically used to estimate onset of active first stage for cases where partograph data of 5 cm was missing but information on cervical dilation before and after 5 cm was documented in the partograph. Each woman with a recorded data timepoint for cervical dilation pre and post 5 cm were subject for imputation, information about the imputation extensively described in^2^ (Figure S1). This method was applied to reduce selection bias in the study population and this has been used in previous publication, published elsewhere^26^ From the Target population we defined the complete case cohort (n = 31,351) as only including observations with a notation of cervical dilation of 5 cm. The final Study cohort included the complete cases plus “15,241” cases with the imputation of the onset of the active first stage with using information from at least 1 notation of cervical dilation < 5 cm and another between 6 and 10 cm, of which, 1% cases (n = 552) do not have documented notation of the end of active phase that were therefore removed from the main analysis in the Study Cohort (Fig. 1).

### Outcomes

Adverse neonatal outcomes were retrieved from the SNQ register and categorized hierarchically into two separate composite adverse neonatal outcomes, *severe* and *moderate.* Severe neonatal outcomes were defined as a composite of neonatal outcome conditions with a high risk of death and/or major neurodevelopmental impairments. Moderate neonatal outcomes were defined as a composite of neonatal outcome conditions with a low risk of death or major impairments if adequate treatment is administered within an appropriate time frame (Table S1 and Table S2).

### Covariates

We selected potential confounding variables based on biological plausibility, a priori knowledge and by a Directed Acyclic Graph (DAG)^27^. Early pregnancy BMI, maternal age^28^ and gestational week were selected as confounders in the final analysis. Gestational week at birth was used as a proxy for birthweight^13,29^. Other covariates considered and tested in the initial analysis (but removed in the final analysis to avoid over adjustment, in line with the framework of DAG) as confounders were hypertensive disease (gestational hypertension, and preeclampsia), pre-gestational diabetes, gestational diabetes, epidural analgesia, fetal position, birthweight and oxytocin use during first stage (Fig. S3).

### Main analysis

Descriptive statistics of baseline characteristics were stratified into four categories (c1-c4), based on the distribution of the active first stage duration (Table 1). Continuous variables were transformed into categorical variables. Our primary independent variable was the duration of the active first stage, and to explore how duration-category were associated with neonatal outcomes, we created four categories of duration of the active first stage by increasing distribution intervals, namely; c1: < 50th percentile (median), c2: 50th to 75th percentile, c3: > 75th to 90th percentile and c4: > 90th percentile (Table 1).

**Table 1:** Maternal and delivery characteristics, categorize into percentiles of first stage duration for the Study cohort including 46 040 women.

	Total N (%)	N (column-%) by active first stage duration (hours)			
		Category 1 (c1): < 50th percentile, < 5.1 h	Category 2 (c2): 50th to 75th percentile, 5.1–7.5 h	Category 3 (c3): > 75th to < 90th percentile, 7.5- < 10.1 h	Category 4 (c4): > 90th percentile, > 10.1 h
Age, years					
≤ 19	826 (1.8)	505 (2.2)	189 (1.7)	83 (1.1)	49 (1.1)
20–24	359 (20.3)	5288 (23.2)	2234 (19.5)	1195 (16.5)	642 (14.2)
25–29	17 480 (38.0)	8643 (38.0)	4317 (37.7)	2826 (38.9)	1694 (37.4)
30–34	13 848 (30.1)	6331 27.8)	3585 (31.3)	2353 (32.4)	1579 (34.9)
35+	4505 (9.8)	2017 (8.9)	1116 (9.8)	807 (11.1)	565 (12.5)
Missing	22 (0.05)	15 (0.07)	5 (0.04)	1 (0.01)	1 (0.02)
Co-habitation					
Yes	41 415 (90.0)	20 365 (90.3)	10 304 (90.0)	6610 (91.0)	4136 (91.3)
No	1459 (6.0)	1459 (6.4)	677 (5.9)	397 (5.5)	228 (5.0)
Other	1062 (2.3)	587(2.6)	248 (2.2)	142 (2.0)	85 (1.9)
Missing	802 (1.7)	388 (1.7)	217 (1.9)	116 (1.6)	81 (1.8)
Body mass index, kg/m^2^					
≤ 25	32 234 (70.0)	16 194 (71.0)	8014 (70.0)	4993 (68.7)	3033 (67.0)
25–29.9	8752 (19.0)	4190 (18.4)	2158 (18.9)	1438 (19.7)	966 (21.3)
30–34.9	2439 (5.3)	1151 (5.0)	592 (5.2)	431 (5.9)	266 (5.9)
35+	785 (1.7)	366 (1.6)	201 (1.8)	135 (1.8)	83 (1.8)
Missing	1830 (3.9)	899 (49.1)	481(4.2)	268 (3.7)	182 (4.0)
Epidural use					
Yes	39 030 (84.7)	17 917 (78.6)	10 204 (89.1)	6649 (91.5)	4260 (94.0)
No	7010 (15.2)	4882 (21.4)	1242 (10.9)	616 (8.5)	270 (6.0)
Oxytocin use					
First stage	27 296 (59.3)	9458 (41.5)	7555 (66.0)	5995 (82.5)	4288 (94.6)
Second stage	8958 (19.5)	5570 (24.4)	2361 (20.6)	865 (11.9)	162 (3.6)
Birthweight, g					
< 3000	5415 (11.8)	3541 (15.5)	1137 (9.93)	510 (7.0)	227 (5.0)
3000–3500	18 036 (39.2)	9873 (43.3)	4376 (38.2)	2471 (34.0)	1316 (29.1)
3501–4000	16 665 (36.2)	7388 (32.4)	4372 (38.2)	3000 (41.3)	1905 (42.1)
4001–4499	5177 (11.2)	1775 (7.9)	1353 (11.8)	1127 (15.5)	922 (20.4)
> 4500	718 (1.6)	207 (0.9)	203 (1.8)	154 (2.1)	154 (3.4)
Missing	29 (0.06)	15 (0.07)	5 (0.04)	3 (0.04)	6 (0.13)
Second stage duration minutes, N =		22,788	11,443	7262	4530
Median, (IQR)		84 (45, 152)	121 (65, 195)	134 (75, 209)	141 (79, 214)
5th, 95th percentile		19,262	26,295	30,306	31,322
Adverse neonatal outcomes, absolute risks					
Severe	979 (2.1)	432 (1.9)	241 (2.1)	171 (2.4)	135 (3.0)
Moderate	1712 (3.7)	627 (2.8)	460 (4.0)	343 (4.7)	282 (6.2)
No adverse outcome	43,349 (94.2)	21,740 (95.4)	10,745 (93.9)	6751 (92.9)	4113 (90.8)
Mode of birth					
Spontaneous vaginal delivery	36 381 (79.0)	19 534 (85.7)	8903 (77.8)	5207 (71.7)	2737 (60.4)
Cesarean delivery (second stage)	1985 (4.3)	382 (1.68)	490 (4.3)	518 (7.1)	595 (13.3)
Operative delivery	7674 (16.7)	2883 (12.7)	2053 17.9)	1540 (21.2)	1198 (26.5)

**Figure 2:**
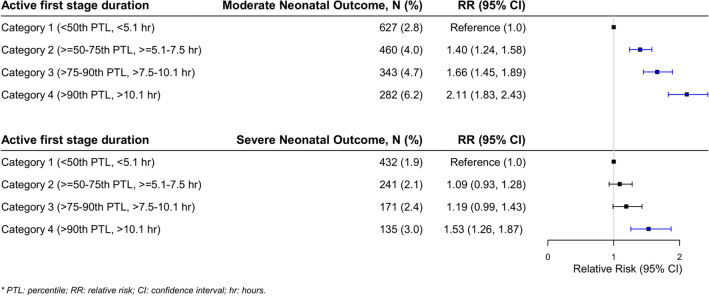
The absolute risk and proportions for severe or moderate neonatal outcomes for the association between increasing active first stage of labour duration presented along with the adjusted relative risk of severe or moderate neonatal outcomes in the forest plot**.** Forest plot for the association between increasing active first stage of labour duration and the relative risk of either severe or moderate neonatal outcome, adjusted relative risk presented.

**Figure 3:**
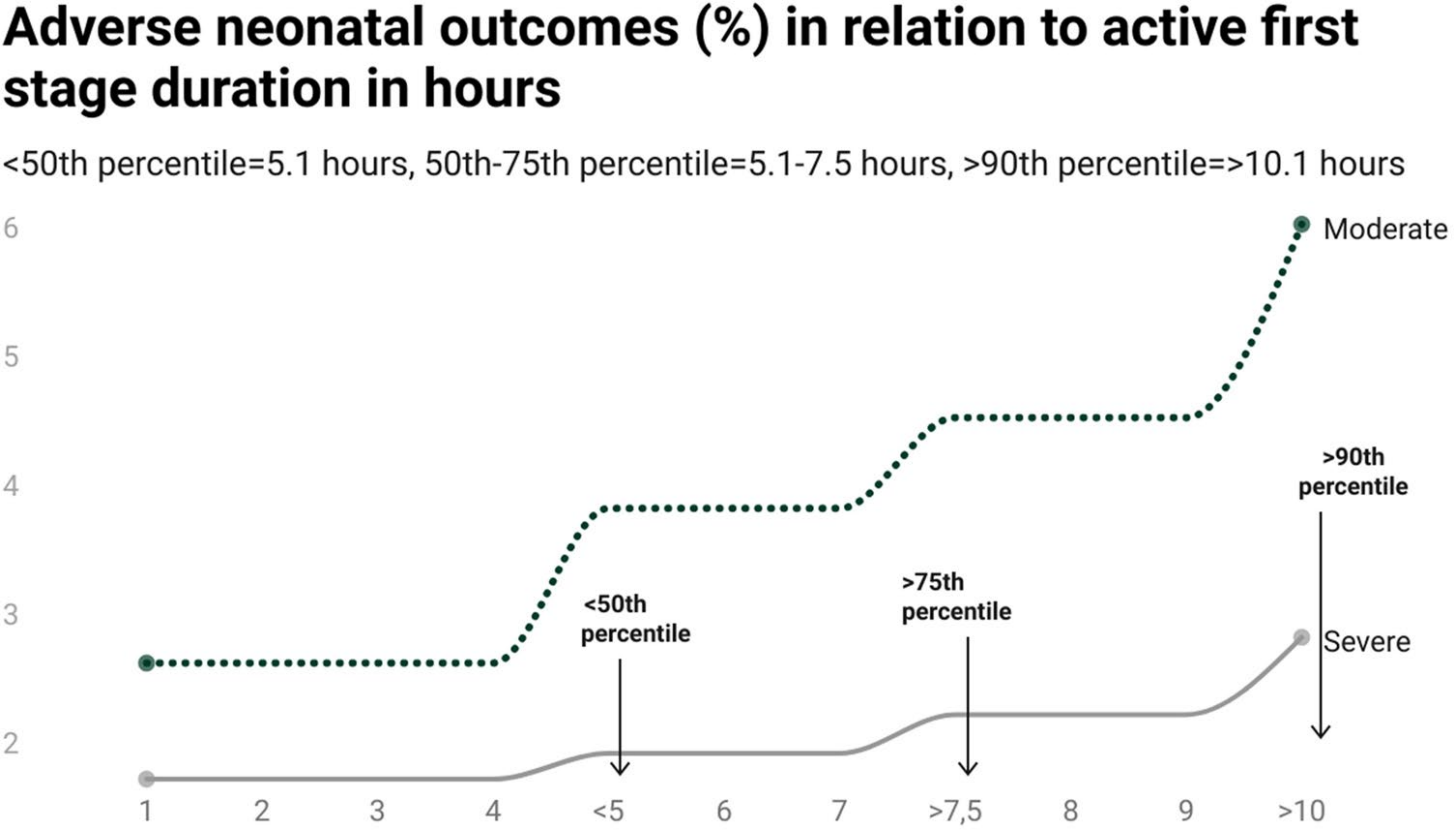
Stratified adverse neonatal outcomes depicted linearly in relation to the active first stage of labour duration for the points of investigated percentiles in the Study Cohort of 46 040 women. Y axis corresponding to absolute risks in % and X corresponds to the continuous exposure “active first stage of labour duration”, categorized.

Poisson regression with robust standard errors was applied to investigate the association between active first stage of labour duration and the relative risk of either severe or moderate neonatal outcome. In the regression analysis, c1 was used as reference. Results are presented as crude and adjusted relative risks with corresponding 95% confidence intervals (CI) (Table 3 and Figs. 2, 3). There were few missing observations on covariates (< 4%), and no imputation methods were applied to them. SAS version 9.4 (SAS Institute Inc., Cary, NC, USA) was used for data management and analyses (Table 2).

**Table 2:** Descriptive data on the duration of active first stage of labour in hours in the Study cohort and the Complete case cohort (reported as percentiles) and stratified by adverse neonatal outcome (Severe, Moderate, No adverse).

Cohort selection	N	p5	p10	p 25	p50	p75	p90	p95
Point of estimated distribution of duration stratified by adverse neonatal outcome duration in hours at below percentiles								
Study cohort	46,040	1.4	2.0	3.2	5.1	7.5	10.1	11.8
Stratified by neonatal outcome								
Severe	979	1.6	2.3	3.5	5.7	8.4	11.0	12.8
Moderate	1712	2.1	2.7	4.2	6.2	8.9	11.2	13.2
No adverse	43,349	1.1	1.9	3.2	5.1	7.5	10.0	11.7
Complete case cohort	31,351	1.6	2.2	3.6	5.6	8.1	10.4	12.2
Stratified by neonatal outcome								
Severe	679	1.9	2.6	4.0	6.2	8.8	11.1	12.9
Moderate	1201	2.4	3.0	4.6	6.6	9.2	11.6	13.8
No adverse	29,471	1.6	2.2	3.6	5.6	8.0	10.4	12.1

### Mediation analysis

A mediation analysis was performed to investigate the extent to which associations between first stage and adverse neonatal outcomes were mediated by second stage of labour duration (Table 4, Figs. S2, S3)^16–18^. This approach, extensively described elsewhere, builds on a counterfactual framework^30–34^. Total effect decomposes into the product of the direct effect and mediated effect, and we also estimated the proportion mediated on the risk difference scale. Here, the mediator, second stage, was modelled both as binary and as a continuous variable (Fig. S2, Table 4), following the significant findings in the main analysis (Table 3). We used the CAUSALMED procedure in SAS for all estimates.

**Table 3:** Active first stage of labour duration and relative/absolute risks of adverse neonatal outcomes in the Study cohort, using the first category (< 50th percentile) of active first stage of labour duration as the reference.

	Severe RR^a^	Severe aRR ^b^		Moderate RR^a^	Moderate aRR ^b^
Active first stage duration			Active first stage duration		
Categorized labour duration and risks of adverse neonatal outcomes in the Study cohort					
Category 1 (c1): < 50th percentile < 5.1 h	Reference	Reference	Category 1 (c1): < 50th percentile < 5.1 h	Reference	Reference
Category 2 (c2): 50th to 75th percentile 5.1–7.5 h	1.13 (0.96, 1.32)	1.09 (0.93, 1.28)	Category 2 (c2): 50th to 75th percentile 5.1–7.5 h	1.46 (1.30, 1.65) *	1.40 (1.24, 1.58) *
Category 3 (c3): > 75th to 90th percentile > 7.5–10.1 h	1.27 (1.06, 1.51) *	1.19 (0.99, 1.43) *	Category 3 (c3): > 75th to 90th percentile > 7.5–10.1 h	1.72 (1.51, 1.96) *	1.66 (1.45, 1.89) *
Category 4 (c4): > 90th percentile > 10.1 h	1.63 (1.35, 1.97) *	1.53 (1.26, 1.87) *	Category 4 (c4): > 90th percentile > 10.1 h	2.28 2.00, 2.62) *	2.11 (1.83, 2.43) *

^a^RR = Relative Risk ^b^adjusted for BMI, Maternal age, Gestational week categorized *Significant, *P*-value < 0.001. *Severe* outcomes are removed from the comparison group for *moderate* outcome. *Moderate* outcomes are removed from the comparison group for *severe* outcome.

**Table 4:** Mediation analysis for comparing increasing active first stage labour’s association to adverse neonatal outcomes; effect of active first stage of labour duration (Total effect TE), mediated through the causal mediator, second stage labour duration, (Natural indirect effect NIE).

Odds ratio (95% CI)									
No	Exposure: Active first stage duration	Causal mediator: Second stage duration	Adverse neonatal outcome	Adjusted (Yes/No)	Total effect (TE)	Controlled direct effect (CDE)	Natural direct effect (NDE)	Natural indirect effect (NIE)	Percentage mediated (95% CI)
1	Binary: duration of active first stage < median (5.15 h) vs ≥ median	Binary: duration of second stage < 2 h vs ≥ 2 h	Moderate	No	1.72 (1.55, 1.91)	1.60 (1.39, 1.86)	1.63 (1.46, 1.81)	1.06 (1.03, 1.08)	13.24 (7.26, 19.42)
2	Binary: duration of active first stage < median (5.15 h) vs ≥ median	Binary: duration of second stage < 2 h vs ≥ 2 h	Moderate	Yes*	1.51 (1.34, 1.68)	1.39 (1.18, 1.62)	1.43 (1.26, 1.60)	1.06 (1.03, 1.09)	**16.14 (8.82, 25.60)**
3	Binary: duration of active first stage < median (5.15 h) vs ≥ median	Continuous: duration of second stage**	Moderate	No	1.72 (1.56, 1.90)	1.59 (1.43, 1.76)	1.59 (1.43, 1.76)	1.08 (1.05, 1.11)	18.01 (11.20, 24.41)
4	Binary: duration of active first stage < median (5.15 h) vs ≥ median	Continuous: duration of second stage	Moderate	Yes*	1.51 (1.35, 1.68)	1.40 (1.25, 1.57)	1.40 (1.25, 1.57)	1.08 (1.05, 1.11)	**20.99 (12.80, 30.77)**
5	Binary: duration of active first stage < 90th percentile (10.07 h) vs ≥ 90th	Continuous: duration of second stage	Severe***	No	1.49 (1.22, 1.80)	1.42 (1.17, 1.72)	1.42 (1.17, 1.72)	1.05 (1.02, 1.08)	13.82 (6.45, 28.34)
6	Binary: duration of active first stage < 90th percentile (10.07 h) vs ≥ 90th	Continuous: duration of second stage	Severe	Yes*	1.37 (1.09, 1.64)	1.32 (1.05, 1.59)	1.32 (1.05, 1.59)	1.04 (1.02, 1.06)	**13.60 (5.49, 39.43)**

Presented as Odds ratios and percentage mediated (%). The causal mediator was modelled both as binary (Model 1–2) and continuous variable (3–6).

CI, confidence interval. The 95% CIs were estimated based on the bias-corrected bootstrap resampling method with 2000 replications. Proportion mediated with a wider CI for severe adverse neonatal outcomes, more data would yield a more precise interval estimate.

*** For the rare severe neonatal outcome and rare exposure (only 10% sample size being exposed), no interaction between the binary exposure and the continuous mediator was added in this analysis.

Significant values are in bold.

### Sensitivity analysis

We performed several sensitivity analyses. First, to account for the possible influence of maternal co-morbidity, we tested several potential confounders in the adjusted analysis. Second, to verify the categorization (percentiles of first stage duration) of our exposure, we reran analyses of relative risks using alternative exposure categorization groups (Table S3). We also investigated the results using first timepoint of contractions as start of the exposure, this to compare if patterns of duration between the outcomes (severe, moderate, no adverse) was robust when using another starting point (Table S4). Third, to further evaluate the robustness of the imputed timepoints for 5 cm, all analyses were also performed in the “Complete case cohort” (Table S5). Four, to examine the potential influence of oxytocin use during first and/or second stage of labour, we performed adjusted analysis including oxytocin as a confounder, additionally we performed an adjusted analysis including also birthweight in the adjusted model. (Table S6). The last sensitivity analysis was performed to describe the distribution of duration and numbers of excluded cases of adverse neonatal outcomes due to caesarean delivery during the active first stage (Fig. 1, Table S7).

### Details of ethical approval

Permission for this study was obtained from Swedish Ethical Review Authority (In Swedish: Etikprövningsmyndigheten, Sweden, http://etikprovningsmyndigheten.se) and approved by the regional ethical committee (IRB) at Karolinska Institutet, Stockholm No 2019-02818, 2009/275-31. The studies in the project are based on previously collected medical record and register data and the personal identification numbers has been replaced by anonymous serial numbers by the Swedish National Board of Health and Welfare. Analyses were conducted on de-identified data and informed consent was collected from caregivers, in accordance with the ethical approvals. All methods were performed in accordance with relevant guidelines and regulations.

## Results

### Main analysis

Among the 46,040 women in the Study cohort, 94.2% experienced a delivery with normal neonatal outcomes. We identified 979 (2.1%) deliveries with a severe outcome, and 1712 (3.7%) with a moderate outcome (Table 1). The median duration in the Study Cohort was 5.1 h. Among deliveries with severe neonatal outcomes the median duration was 5.7 h and among moderate neonatal outcomes the median was 6.2 h. Absolute risk for moderate outcomes increased from 2.8% (c1) to 6.2 (c4), and absolute risk for severe outcomes from 1.9% (c1) to 3.0% (Table 1, Table 3, Figs. 2, 3). With increasing active first stage duration, the proportions of women with a caesarean delivery during second stage increased, from 4.3% (< 50th percentile, c1) to 13.3% (> 90th percentile, c2) (Table 1).

There was an association between increasing active first stage duration and the relative risk of adverse neonatal outcomes which persisted in the adjusted analysis for moderate neonatal outcomes for all duration categories (c2-c4) compared to the reference (c1). Compared to women with an active first stage < 5.1 h (c1), women with an active first stage of labour between 5.1 and 7.5 h (c2) had an increased risk of moderate adverse neonatal outcomes (aRR 1.4, 95% CI 1.24, 1.58). The most pronounced relative risk was observed among women with an active first stage beyond 10.1 h (c4) compared to women in (c1) (aRR 2.11, 95% CI 1.83, 2.43) (Figs. 2, 3, Table 3).

Increasing labour duration prior to 10.1 h was not associated with an increased relative risk of severe outcomes to the same extent as for the moderate neonatal outcomes. However, when comparing women with labours beyond 10.1 h (c4) to women with < 5.1 h (c1), the relative risk of severe neonatal outcomes was 1.53 (95% CI 1.26, 1.87) (Figs. 2, 3, Table 3).

### Results from mediation analysis

We then assessed the role of second stage duration on the association between active first stage duration and adverse neonatal outcomes (Fig. S2). When modelling second stage as a continuous mediator, approximately 21% of the total effect was mediated through the duration of second stage (Table 4, model 4).

For severe neonatal outcomes, among women with an active first stage duration > 10.1 h versus less than 10.1 h, 14% of the total effect was mediated through the second stage duration (Table 4, model 6). To conclude, this analysis showed that a most of the risk on adverse neonatal outcomes were due to active first stage duration since a smaller percentage (21% and 14% respectively) was related to the indirect effect of an increasing second stage duration.

### Results from sensitivity analyses

In a sensitivity analysis, neither hypertensive disease, gestational hypertension or preeclampsia nor gestational diabetes significantly altered the point estimates for risk of adverse neonatal outcomes and were therefore not included as confounders in the final analysis. In the second sensitivity analysis, as expected the results were marginally changed (Table S3). Same pattern was also observed when we tested a different start of the exposure (Table S4). Sensitivity analysis performed in the Complete case cohort, showed that results were consistent with findings from the main cohort (Table S5). Including oxytocin and birthweight as a confounder only marginally reduced the risk estimates (Table S6). In our last sensitivity analysis, we performed a restricted analysis for women experiencing an adverse neonatal outcome and not included in the population due to a caesarean delivery during first stage. They had slightly shorter overall active first stage duration at all investigated percentiles. Here it was obvious that a majority of these cases 43.8%, n = 74 (moderate), and 52% n = 31 (severe) showed an early sign of fetal distress, and had a caesarean delivery before 5.1 h (50th percentile) (Table S7).

## Comment

### Principal findings

Increasing active first stage duration in our population of nulliparous women with term pregnancies and spontaneous onset was associated with increased relative risks of both moderate and severe neonatal outcomes. Effect decomposition further suggested that most of this association was not mediated by second stage of labour duration.

### Results in the context of what is known

These study results contribute to prior findings showing an association between longer active first stage of labour durations and adverse neonatal outcomes in high income settings. Our findings echo previous publications by Blankenship et al.^12^ who reported increasing neonatal morbidity for labours > 11.3 h. Different from ours, their study included parous and induced labours and investigated a slightly different composite neonatal outcome. Similarly, the most recent study from the Consortium on Safe Labour (based on data from 2002 to 2008) concluded that slower labour (≥ 4 h) at 6–7 cm was not associated with risk but allowing arrest of dilation of ≥ 4 h between 8 and 9 cm increased the risk for adverse neonatal outcomes^6^. Considered together, these findings suggest that increasing active first stage of labour duration, rather than a strict duration threshold at a specific cervical dilation, may increase risk.

Creating composite neonatal outcomes is not straightforward. Some neonatal outcomes are associated with potentially debilitating or life-threatening consequences while others are not. For this reason, we chose to analyses adverse neonatal outcomes indicative of serious concerns for long term consequences separately from those that are less likely to have long term consequences for the neonate, when adequately treated. For example, the underlying etiology for the severe outcome intracranial hemorrhage (ICH) may have been developed antenatally or acquired during labour, however the consequences could be fatal and actions to prevent ICH is important. Although we used a more clinically relevant composite neonatal outcome, our findings appear similar to previous publications^6,7,12,14^, with an important finding that neonates with a composite of severe outcomes had shorter distribution of duration than moderate outcomes.

The mediation analysis addressed the questions: (a) “Is second stage labour duration mediating the association between active first stage of labour and adverse neonatal outcome?” and (b) “Is the mediated effect of second stage of labour duration similar for moderate and severe neonatal outcomes?” Study results indicate that first stage duration is independently associated with adverse neonatal outcomes, given that only 21% and 13% was mediated through second stage duration for severe and moderate outcomes, respectively.

There is conflicting evidence regarding the association between first and second stage of labour duration and adverse neonatal outcomes: some studies report no increased risk^15,35,36^, while others have found that a prolonged duration is associated with increased neonatal morbidity^16,17,20,37,38^. This may be partly explained by the fact that studies addressing the impact of labour duration have used a predefined binary threshold of 95th or 90th percentiles duration (first or second stage) and estimated risks for discrete additional increments in duration (i.e. hours) beyond these thresholds. This may indicate that predefined binary thresholds at 95th and 90th percentiles duration are not clinically meaningful. Also, discordances in the neonatal outcomes investigated hamper comparisons between different studies^1,12–15,17,20,35,38^.

### Clinical and research implications

Our study offers novel findings that challenge the value of using points of statistical distribution to define normal vs. abnormal labour duration threshold, focusing on the continuous process of labour duration. The findings from this study may safeguard against potential unintended fatal consequences of practice change based on average separate thresholds for slow progression during first and second stage of labour. Vice versa, potential unintended consequences of a “better safe than sorry regime” approach needs to be balanced against the risk of long-term consequences for both mother and neonate when terminating labour solely due to ‘slow labour’.

Other factors need also to be considered in the context of studying labour duration; uterine contractions cause intermittent decreases in uteroplacental blood flow, resulting in temporarily reduced blood and oxygen delivery to the fetus^39,40^. Most fetuses tolerate these normal labour processes very well. We hypothesis that other factors such as placental insufficiency, inflammation or sub-clinical infection that, when combined with longer labour, may increase the risk. There may also be some fetuses who are more sensitive to medical practices, such as oxytocin augmentation, higher dosage and time augmented are reported for women with longer duration. By widening our focus beyond labour duration to consider combinations of anthropometrics and medical practices that increase or decrease fetal tolerance for labour, we may ultimately be more successful in defining clinical interventions to identify fetuses who cannot tolerate labour much beyond the median duration vs. fetuses who are impervious to longer durations. These cases are apparent in the sensitivity analysis restricted to the few cases of adverse neonatal outcomes among women with caesarean deliveries due to fetal distress (Fig. 1, Table S7), where the majority of cases were found in the < 50th percentile, 5.1 h.

Studies suggest that identifying a firm, universal labour duration threshold for when neonatal risk increases is challenging and may not be feasible. There is an urgent need to better elucidate the physiological and patho-physiological factors that influence women and neonates during spontaneous onsets to better guide clinicians Incidence rates of adverse neonatal outcomes in term gestations (> 37 weeks)- among low-risk women are low and relative risk or odds ratios commonly used may be difficult to interpret. For these reasons, the absolute neonatal risk related to labour duration may be a more appropriate measure for clinical guidance^12–14^.

### Strengths and limitations

Strengths of our study include the use of a large population-based cohort linked data with the validated SNQ quality register providing the most robust outcome data available^25^. Compared to previous research, this study was adequately powered for comparison at higher percentiles of duration despite the prior known rarity of some of the adverse neonatal outcomes. After imputation for start of active phase for women with missing notation of (first stage of labour), i.e. 5 cm cervical dilation, we were able to be consistent with recommendations for active labour duration research^21,41^. Since we were also able to include women with dilation of 3.4 cm we could reduce both selection bias and misclassification of the exposure, which is the well-established major research challenge in studies investigating duration. A series of sensitivity analysis performed that only slightly changed the results, demonstrating robust results in the final study cohort. Using mediation analysis to quantify the effect of different levels of duration in a broader perspective has to our knowledge not been applied for studies investigating the impact of labour duration on adverse neonatal outcomes^30–33^.

Limitations in our analyses is that our model did not include latent phase duration (a possible confounder) and selection bias introduced by including only women who reached the second stage of labour. The influence of latent phase and the differences in duration between spontaneous onsets in different cohorts has been reported and described elsewhere^2,18^. Importantly, this investigated cohort does not include any women experiencing a caesarean delivery during the active first stage of labour, how labour duration in relation to their neonatal outcomes has not been investigated in this study, with the exemption those included in the sensitivity analysis in Table S7. We did not expand our cohort to include any women with > 5 cm dilation upon arrival, since we could not accurately measure the start of exposure for them, hence it is possible that the study cohort is biased since a few women with faster labours (being dilated more than 5 cm upon arrival) could have been excluded from the cohort. We do not think that that would have influenced our robust estimates, since being dilated more than 5 cm is uncommon for nulliparous women arriving at the delivery wards in Sweden. Mediation analysis was restricted to second stage duration, other potential mediators of interest (augmentation with oxytocin, epidural, mode of delivery) were not investigated. Though we were able to adjust for several confounders and also tested in sensitivity analysis a wide range of covariates, we were unable to control for unknown factors. Residual confounding may also exist to some extent due to how categorization of known confounders was performed. One more limitation is that despite the data were captured from the partograph prospectively, the intention of this study is describing association. Hence, the study is not designed for predictions for at what exact timepoint of duration clinicians should intervene to avoid adverse outcome. The generalizability is restricted to similar populations and settings for spontaneous onsets, where the caesarean delivery rate during first stage of labour is low.

## Conclusions

We report an association between increasing active first stage duration and increased risks of adverse neonatal outcomes. The absolute risk of experiencing an adverse neonatal was low in this study population. Given the findings and the overall high use of interventions to mitigate slow labour, future strategies are needed to identify neonates who are vulnerable to longer labours for targeted intervention strategies and clinical guidance.

## Supplementary Information

Supplementary Figure 1.

Supplementary Figure 2.

Supplementary Figure 3.

Supplementary Tables.

## Data Availability

The Stockholm-Gotland Obstetric Cohort was used for this study. Information in the databased was retrieved from the medical record system Obstetrix. The database is stored in the Unit of Clinical epidemiology at Karolinska Institutet Stockholm, Sweden. Public data sharing from this database is not permitted. However, any research can access the data by obtaining an ethical approval from a regional ethical review board and are available from the corresponding author on reasonable request.
