# Supplementary figures and images for: First stage of labour duration and associated risk of adverse neonatal outcomes
Source: Sci Rep. 2023 Aug 2;13:12569. doi: 10.1038/s41598-023-39480-0 (PMC10397187; doi:10.1038/s41598-023-39480-0)

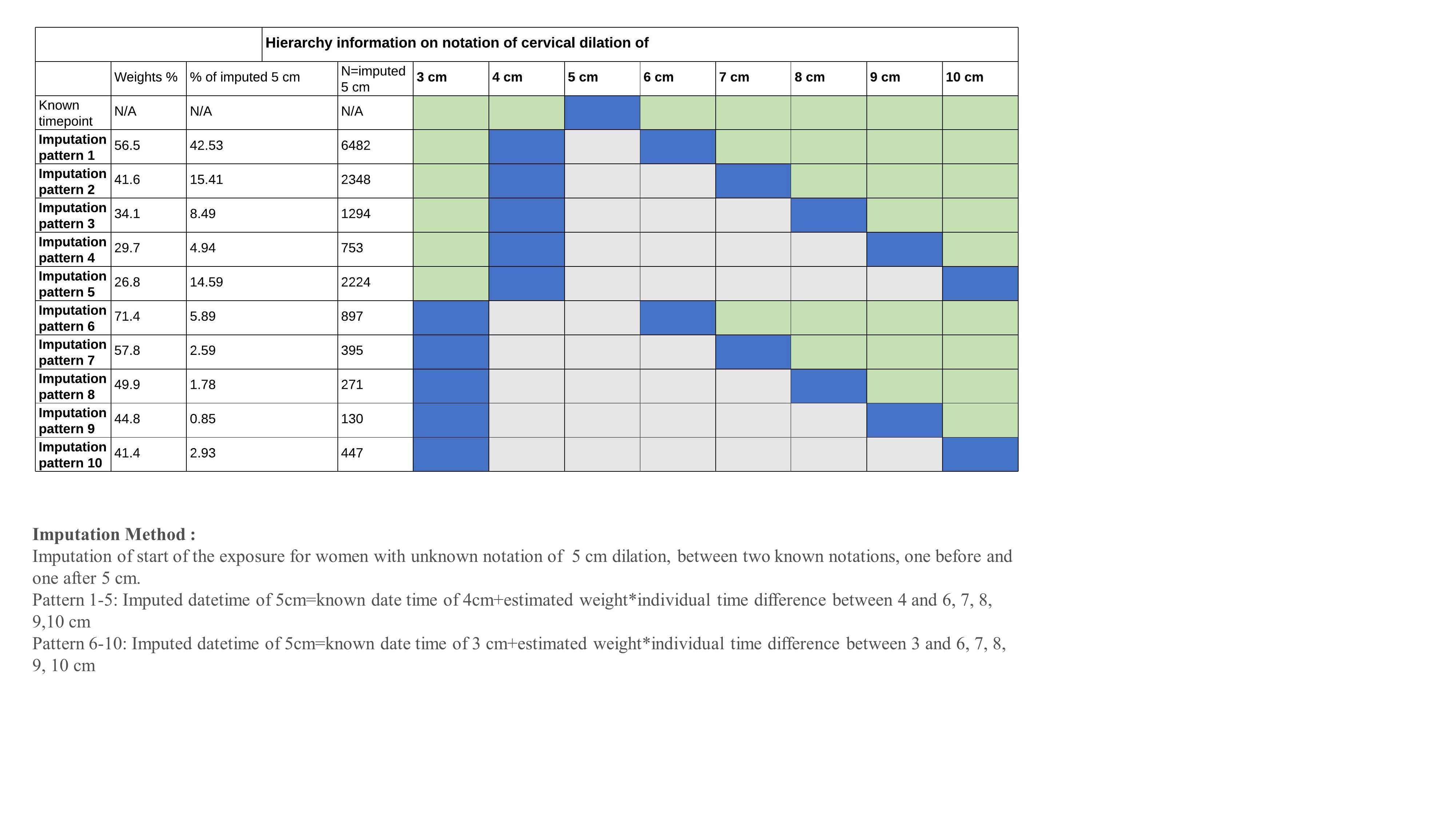

Supplement: Supplementary file 1 — Supplementary Figure 1. [file 41598_2023_39480_MOESM1_ESM.jpg]

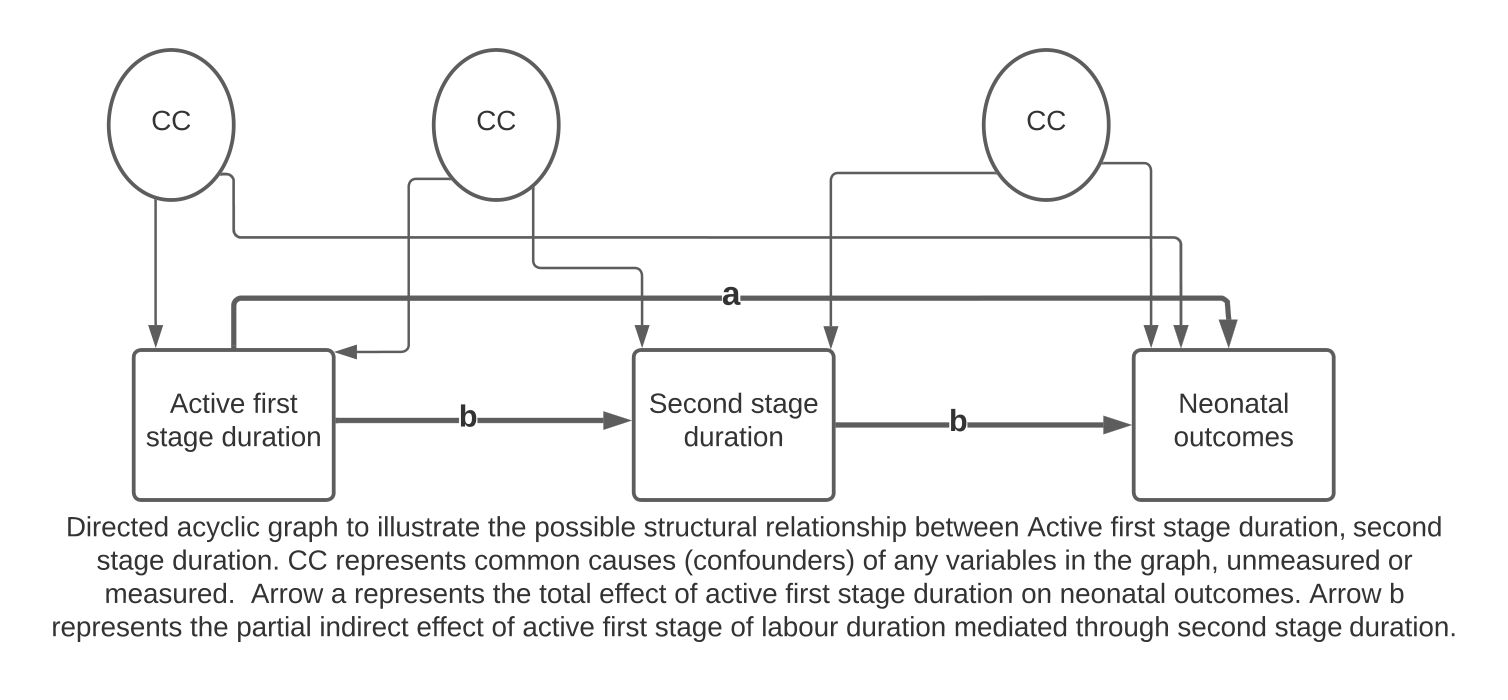

Supplement: Supplementary file 2 — Supplementary Figure 2. [file 41598_2023_39480_MOESM2_ESM.tiff]

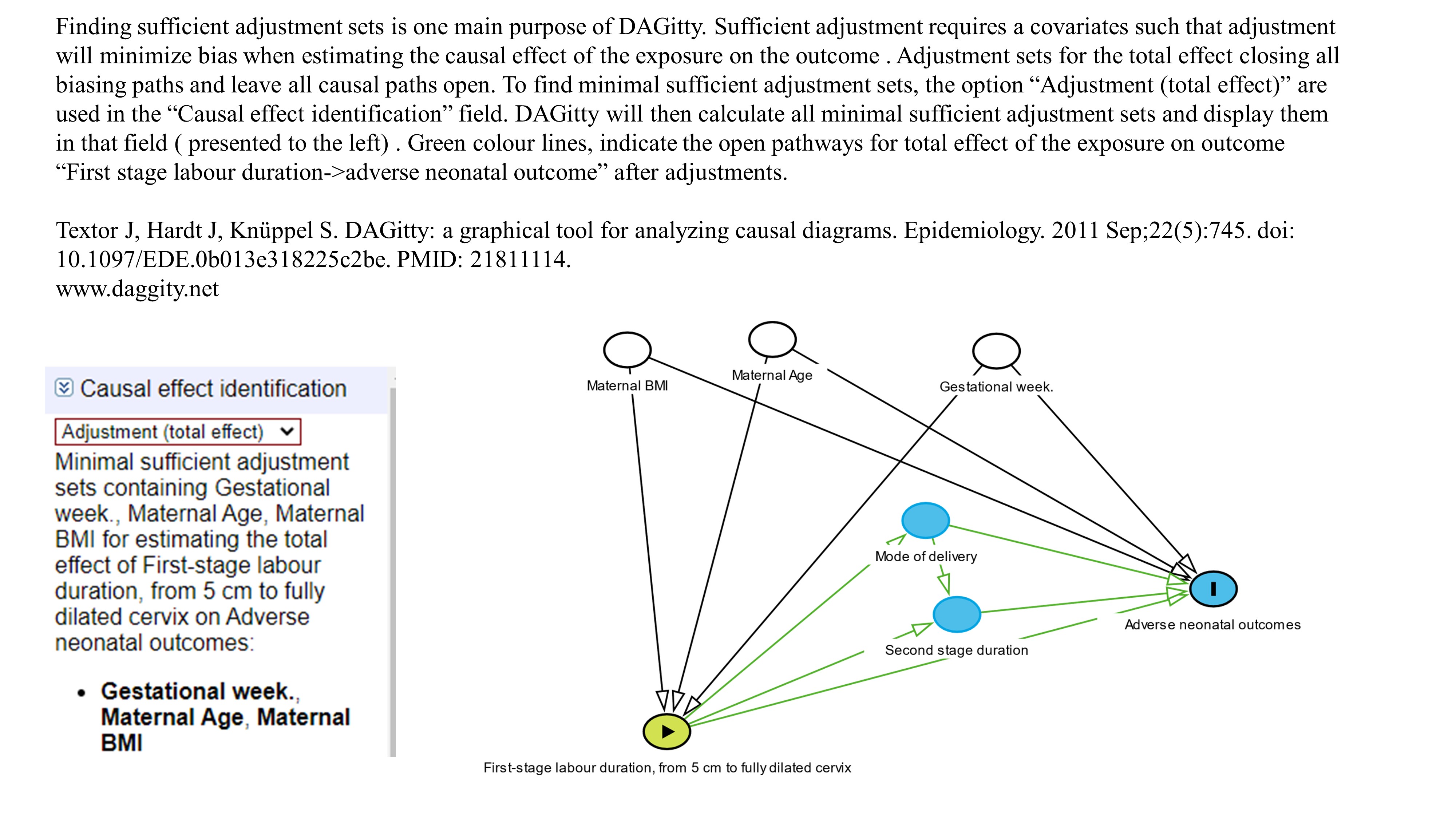

Supplement: Supplementary file 3 — Supplementary Figure 3. [file 41598_2023_39480_MOESM3_ESM.jpg]
