# Supplementary material for: First stage of labour duration and associated risk of adverse neonatal outcomes
Source: Sci Rep. 2023 Aug 2;13:12569. doi: 10.1038/s41598-023-39480-0 (PMC10397187; doi:10.1038/s41598-023-39480-0)
Supplement: Supplementary file 4 — Supplementary Tables. [file 41598_2023_39480_MOESM4_ESM.docx]

| **Table S1.** Detailed definitions for composite outcomes, including rational and reference. | | |
| --- | --- | --- |
| **Conditions** | **Rational** | Reference |
| **Severe** | **Outcomes with a high risk of death and/or major neurodevelopmental impairments, including cerebral palsy, cognitive impairment, visual impairment or hearing impairment.** |  |
| Intrapartum fetal death | Severe since it involves death of the neonate during labour or close after birth, related to labour |  |
| Hypothermia treatment | Severe since hypothermia treatment is only given to infants with fulfilled A-criteria, which corresponds to clinical signs of pronounced asphyxia at birth, and these neonates have a high risk of future major neurodevelopmental impairments (NDI). | ^1, 2^ |
| Birth asphyxia related complications | Severe for infants with pronounced asphyxia, since it is well known that they have a risk of NDI. (P.21.0) Severe for infants with moderate to severe HIE indicate an impact of the brains that increases the risk of NDI. Therapeutic hypothermia reduces this risk but does not remove it | ^1, 3^ |
| Neonatal seizures | Seizures are the most frequent manifestation of NDI disorder in the new-born period, of course the risk of NDI is depending on the aetiology behind the seizure, but at large there is an increased risk of NDI and death and therefore it is appropriate to classify it as severe outcome | ^4^ |
| Chest compressions at birth | Severe since it is a sign of severe birth asphyxia. |  |
| Intubation | Sign of or cause to severe birth asphyxia |  |
| APGAR at 5, <4 | A marker for severe birth asphyxia | ^5-7^ |
| Umbilical-artery acidosis Ph<7.00, and/or Base deficit of ≥16 | Severe since it is a sign of severe birth asphyxia | ^2^ |
| Intracranial bleeds | Deep intracerebral bleeding, with a risk of NDI, implies haemorrhagic stroke which has a high risk of NDI.  Notice regarding Intracranial bleeds, no new validated ICD codes chart review has been recently published for other diagnoses (P10.8, P52.0, P.52.9) It may be that some of these children diagnosed with either intracerebral bleedings only have subdural, subarachnoid or minor IVHs which not increase the risk of NDI and are common after normal deliveries. | ^8-11^ |
| Intracranial stroke | High risk of NDI | ^10, 11^ |
| **Moderate** | **Outcomes with a low risk of death and/or major impairments and are less likely to have long term consequences for the infant.** |  |
| Neonatal sepsis | Early diagnosed sepsis, within 72 hours after birth, since we are aiming to investigate effects of labour. This is a less severe condition in a Swedish setting, since sepsis is early detected and adequate treatment is given with wide inclusions and the risk for death and NDI is therefore low in term neonates | ^12, 13^ |
| Quickly recovering indications of asphyxia | Moderate since the infants recover quickly and the risk of mortality/NDI is not high, even though some recent studies have shown that also a slight decrease in Apgar leads to an increased risk of cerebral palsy and epilepsy. Also, a quickly recovering HIE, since these children were considered to become healthy in the majority of cases. Some studies have shown that also these children (on a population level) have slightly lower cognitive score on Bayley’s testing at 2 years compared to healthy children without a HIE diagnose as infant, however they do not seem to have a proven increased risk of death o major NDI, and the absolute risk must be considered very low | ^2^ |
| Pneumothorax | Pneumothorax may lead to acute morbidity and risk of death but in Sweden you would expect that the children get adequate treatment and then the risk of death/NDI should be low. Temporality for this condition is uncertain, could be a consequence of treatment after birth, all cases identified in moderate or severe has at least one another condition which still is allocating them to either category. |  |
| Quickly recovering respiratory disturbances | Moderate since these conditions have a low risk for death/NDI as adequate treatment are given |  |
| APGAR at 5 ≥4-<7 | Moderate since most of these neonates, particularly born at full term recover well. | ^14, 15^ |
| Meconium aspiration | Moderate since most infants with meconium aspiration have a mild-moderate form with low risk of death/NDI. Very severe meconium aspiration is life threatening and if the infant need ECMO it would of course be severe (and have any additional diagnose for severe) , however for meconium aspiration in general, without any other condition diagnosed as severe this categorization is adequate |  |

- 1. Jacobs SE, Berg M, Hunt R, Tarnow-Mordi WO, Inder TE, Davis PG. Cooling for newborns with hypoxic ischaemic encephalopathy. The Cochrane database of systematic reviews. 2013 Jan 31;2013(1):Cd003311.
- 2. Papile LA, Baley JE, Benitz W, Cummings J, Carlo WA, Eichenwald E, et al. Hypothermia and neonatal encephalopathy. Pediatrics. 2014 Jun;133(6):1146-50.
- 3. Finder M, Boylan GB, Twomey D, Ahearne C, Murray DM, Hallberg B. Two-Year Neurodevelopmental Outcomes After Mild Hypoxic Ischemic Encephalopathy in the Era of Therapeutic Hypothermia. JAMA pediatrics. 2020 Jan 1;174(1):48-55.
- 4. Uria-Avellanal C, Marlow N, Rennie JM. Outcome following neonatal seizures. Seminars in fetal & neonatal medicine. 2013 Aug;18(4):224-32.
- 5. Cnattingius S, Norman M, Granath F, Petersson G, Stephansson O, Frisell T. Apgar Score Components at 5 Minutes: Risks and Prediction of Neonatal Mortality. Paediatric and perinatal epidemiology. 2017 Jul;31(4):328-37.
- 6. Casey BM, McIntire DD, Leveno KJ. The continuing value of the Apgar score for the assessment of newborn infants. The New England journal of medicine. 2001 Feb 15;344(7):467-71.
- 7. Iliodromiti S, Mackay DF, Smith GC, Pell JP, Nelson SM. Apgar score and the risk of cause-specific infant mortality: a population-based cohort study. Lancet (London, England). 2014 Nov 15;384(9956):1749-55.
- 8. Cole L, Dewey D, Letourneau N, Kaplan BJ, Chaput K, Gallagher C, et al. Clinical Characteristics, Risk Factors, and Outcomes Associated With Neonatal Hemorrhagic Stroke: A Population-Based Case-Control Study. JAMA pediatrics. 2017 Mar 1;171(3):230-8.
- 9. Tan AP, Svrckova P, Cowan F, Chong WK, Mankad K. Intracranial hemorrhage in neonates: A review of etiologies, patterns and predicted clinical outcomes. European journal of paediatric neurology : EJPN : official journal of the European Paediatric Neurology Society. 2018 Jul;22(4):690-717.
- 10. Aberg K, Norman M, Pettersson K, Jarnbert-Pettersson H, Ekeus C. Protracted vacuum extraction and neonatal intracranial hemorrhage among infants born at term: a nationwide case-control study. Acta obstetricia et gynecologica Scandinavica. 2018 Dec 21.
- 11. Walås A, Svensson K, Gyris M, Bang P, Sundelin HEK. Paediatric ischaemic stroke is a valid diagnosis in the Swedish National Patient Register. Acta paediatrica (Oslo, Norway : 1992). 2021 Feb 4.
- 12. Shane AL, Sánchez PJ, Stoll BJ. Neonatal sepsis. Lancet (London, England). 2017 Oct 14;390(10104):1770-80.
- 13. Johansson Gudjónsdóttir M, Elfvin A, Hentz E, Adlerberth I, Tessin I, Trollfors B. Changes in incidence and etiology of early-onset neonatal infections 1997-2017 - a retrospective cohort study in western Sweden. BMC pediatrics. 2019 Dec 12;19(1):490.
- 14. Persson M, Razaz N, Tedroff K, Joseph KS, Cnattingius S. Five and 10 minute Apgar scores and risks of cerebral palsy and epilepsy: population based cohort study in Sweden. BMJ (Clinical research ed). 2018 Feb 7;360:k207.
- 15. Razaz N, Cnattingius S, Persson M, Tedroff K, Lisonkova S, Joseph KS. One-minute and five-minute Apgar scores and child developmental health at 5 years of age: a population-based cohort study in British Columbia, Canada. BMJ open. 2019 May 9;9(5):e027655.

| **Table S2**. Diagnose codes used for outcome definition | |
| --- | --- |
| **Conditions** | **Identified by ICD and/or procedure code in SNQ or Stockholm Gotland obstetric database (OBX)** |
| **Severe** |  |
| Intrapartum fetal death | SNQ/OBX |
| Hypothermia treatment | SNQ /OBX  DV034 |
| Birth asphyxia related complications, severe. | **P21.0**  P91.6  P91.6B  P916.C  P916.X  SNQ/OBX |
| Neonatal seizures | **P90.9**  P90.9A  P90.9B  P90.9C  SNQ/OBX |
| Chest compressions at birth | SNQ/OBX |
| Intubation | SNQ/OBX  DM004 |
| Apgar at 5, <4 | P21.1B  SNQ/OBX |
| Umbilical-artery acidosis  Ph <7.00 or  base excess ≥16 | SNQ/OBX |
| Intracranial bleeds | I61.0  P10.8  P52.0-P52.9  SNQ/OBX |
| Ischemic stroke | I63.0-I63.9  I64.0  SNQ/OBX |
| **Moderate** |  |
| Neonatal sepsis | P36.0-36.5, P36.8  P36.9  P39.2  SNQ/OBX |
| Pneumothorax | P25.1  P25.2  J93.0  J93.1,  J93.8  J93.9  SNQ/OBX |
| Quickly recovering respiratory disturbances | P22.0  P22.1  P22.8  P22.9 |
| Apgar at 5 ≥4-<7 | SNQ/OBX |
| Meconium aspiration | P24.0  SNQ/OBX |

| **Table S3.** Sensitivity analysis, testing a different categorization of the exposure (in hours) and relative risk of adverse neonatal outcomes among the Study cohort of 46 040 women, with first category of active first stage of labour duration as the reference. | | | | | |
| --- | --- | --- | --- | --- | --- |
| **Categorized labour duration and risks of adverse neonatal outcomes in the Study population** | | | | | |
|  | **Severe crude^a^** | **Severe adjusted ^ab^** |  | **Moderate crude^a^** | **Moderate adjusted ^ab^** |
| **Active first stage duration** |  |  | **Active first stage duration** |  |  |
| **S.Category 1:**  <6.1 hours  <60^th^ percentile | Reference (1.0) | Reference (1.0) | **S.Category 1:**  <6.1 hours  <60^th^ percentile | Reference (1.0) | Reference (1.0) |
| **S.Category 2**:  6.1-8.5 hours  60^th^ -≤80^th^ percentile | 1.14 (0.96, 1.32) | 1.09 (0.93, 1.29) | **S.Category 2**:  6.1-8.5 hours  60^th^ -≤80^th^ percentile | 1.34 (1.19, 1.50) ^*^ | 1.27 (1.25, 1.43) |
| **S.Category 3:**  8.5-11.1 hours  80^th-^≤90^th^ percentile | 1.41 (1.18, 1.70)^*^ | 1.34 (1.11, 1.62)^*^ | **S.Category 3:**  8.5-11.1 hours  80^th-^≤90^th^ percentile | 1.95 (1.71, 2.21) ^*^ | 1.85 (1.63, 2.11) ^*^ |
| **S.Category 4:**  >11.1 hours  >90^th^ percentile | 1.60 (1.28, 1.99) ^*^ | 1.47 (1.17, 1.85) ^*^ | **S.Category 4**:  >11.1 hours  >90^th^ percentile | 1.98 (1.69, 2.32) ^*^ | 2.11 (1.83, 2.43) ^*^ |
| **^a^** relative risk, ^b^ adjusted for BMI, Maternal age, Gestational week categorized, ^*)^ Significant, P-value < 0.001. *Severe* outcomes are removed from the comparison group for *moderate* outcome. *Moderate* outcomes are removed from the comparison group for *severe* outcome. | | | | | |

| **Table S4.** Sensitivity analysis, testing a different start of the exposure (duration) to investigate patterns of duration in relation to the outcome, descriptive data on the duration of Total duration (reported as percentiles) and stratified by adverse neonatal outcome (Severe, Moderate, No adverse). In this model, self-reported timepoint for labour onset was used as start of duration and end of exposure period was end of first stage, fully dilated cervix). | | | | | | | | |
| --- | --- | --- | --- | --- | --- | --- | --- | --- |
| **Point of distribution of estimated distribution of Total duration in hours** | | | | | | | | |
| **Cohort selection** | N | p5 | p10 | p 25 | p50 | p75 | p90 | p95 |
| **Total duration** | 85851 | 6.0 | 8.0 | 12.4 | 18.8 | 26.5 | 35.1 | 40.1 |
| ***Stratified by neonatal outcome:*** | | | | | | | | |
| **Severe** | 1665 | 5.9 | 7.8 | 12.2 | 18.7 | 29.0 | 35.0 | 40.0 |
| **Moderate** | 2848 | 7.9 | 9.4 | 14.1 | 20.6 | 28.1 | 37.1 | 41.4 |
| **No adverse** | 81 338 | 5.9 | 7.9 | 12.3 | 18.7 | 26.4 | 35.1 | 40.1 |

| **Table S5.** Active first stage labour duration and risk of adverse neonatal outcomes among the Complete case cohort of 31,351 women, using the first category of active first stage of labour duration as the reference. | | | | | |
| --- | --- | --- | --- | --- | --- |
| **Categorized labour duration and relative risks of adverse neonatal outcomes in Complete case cohort** | | | | | |
|  | **Severe crude^a^** | **Severe adjusted ^ab^** |  | **Moderate crude^a^** | **Moderate adjusted ^ab^** |
| **Active first stage duration** |  |  | **Active first stage duration** |  |  |
| **Category 1** (c1):  <50^th^ percentile  <5.6 hours | Reference (1.0) | Reference (1.0) | **Category 1** (c1):  <50^th^ percentile  <5.6 hours | Reference (1.0) | Reference (1.0) |
| **Category 2** (c2):  50^th^ to 75^th^ percentile  5.6-8.1 hours | 1.14 (0.95, 1.37) | 1.12 (0.93, 1.35) | **Category 2** (c2):  50^th^ to75^th^ percentile  5.6-8.1 hours | 1.30 (1.23, 1.49) ^*^ | 1.24 (1.07, 1.44) ^*^ |
| **Category 3** (c3):  >75^th^ -≤90^th^ percentile  8.1 to10.4 hours | 1.35 (1.01, 1.67) ^*^ | 1.29 (1.04, 1.60)^*^ | **Category 3** (c3):  >75^th^ -90^th^ percentile  8.1 to 10.4 hours | 1.80 (1.54, 2.09) ^*^ | 1.73 (1.48, 2.01) ^*^ |
| **Category 4** (c4):  >90^th^ percentile  >10.4 hours | 1.56 (1.24, 1.97) ^*^ | 1.47 (1.15, 1.87) ^*^ | **Category 4** (c4):  >90^th^ percentile  >10.4 hours | 2.10 (1.78, 2.48) ^*^ | 1.93 (1.63, 2.29) ^*^ |
| **^a^** relative risk, ^b^ adjusted for BMI, Maternal age, Gestational week categorized, ^*)^ Significant, P-value < 0.001. *Severe* outcomes are removed from the comparison group for *moderate* outcome. *Moderate* outcomes are removed from the comparison group for *severe* outcome | | | | | |

| **Table S6.** Sensitivity analysis. Labour duration adjusted risk of adverse neonatal outcomes in Study cohort with *additional adjustments for oxytocin*, model 1 and *additional adjustments for birthweight,* model 2 | | | | | |
| --- | --- | --- | --- | --- | --- |
| **Categorized labour duration and relative risks of adverse neonatal outcomes** | | | | | |
|  | **Model 1** | |  | **Model 2** | |
|  | **Moderate Adjusted ^ab^** | **Severe Adjusted ^ab^** |  | **Moderate Adjusted ^ab^** | **Severe Adjusted ^ab^** |
| **Active first stage duration** |  |  | **Active first stage duration** |  |  |
| **Category 1** (c1):  <50^th^ percentile  <5.6 hours | Reference (1.0) | Reference (1.0) | **Category 1** (c1):  <50^th^ percentile  <5.6 hours | Reference (1.0) | Reference (1.0) |
| **Category 2** (c2):  50^th^ to 75^th^ percentile  5.6 -8.1 hours | 1.40 (1.24, 1.58) ^*^ | 1.07 (0.91, 1.26) | **Category 2** (c2):  50^th^ to75^th^ percentile  5.6-8.1 hours | 1.47 (0.99, 1.33) | 1.09 (0.90, 1.33) |
| **Category 3** (c3):  >75^th^ to90^th^ percentile  8.1-10.4 hours | 1.66 (1.45, 1.88) ^*^ | 1.15 (0.94, 1.39) | **Category 3** (c3):  >75^th^ to90^th^ percentile  8.1-10.4 hours | 1.55 (1.32, 1.82) ^*^ | 1.22 (0.99, 1.55) |
| **Category 4** (c4):  >90^th^ percentile  >10.4 hours | 2.11 (1.83, 2.43) ^*^ | 1.46 (1.18, 1.80) | **Category 4** (c4):  >90^th^ percentile  >10.4 hours | 1.70 (1.41, 2.04) ^*^ | - 1. (1.09, 1.81) |

| **Table S7.** Sensitivity analysis, restricted to excluded women with any adverse neonatal outcomes after start of the exposure, 5 cm dilation and not included in the Complete case cohort due to a caesarean delivery before fully dilated cervix (end of the exposure period). | | | | | | | | | | |
| --- | --- | --- | --- | --- | --- | --- | --- | --- | --- | --- |
|  | **N** | **Point of estimated distribution of duration stratified by adverse neonatal outcome, calculated from start of active of first stage until birth by caesarean delivery** | | | | | | | | |
|  |  | **5th percentile** | **10^th^ percentile** | | **25^th^ percentile** | **Median duration** | | **75^th^ percentile** | **90^th^ percentile** | **95^th^ percentile** |
| **Moderate** | 169 | 1.22 | 1.81 | | 3.17 | 5.83 | | 9.42 | 13.28 | 15.98 |
| **Severe** | 59 | 0.52 | 0.85 | | 2.22 | 4.92 | | 8.80 | 13.73 | 14.53 |
| ***Stratified on Active first stage duration categorized, the categorization is identical to the categorization done for the Complete case cohort, to identify when during the active phase adverse cases with any of these outcomes are delivered with caesarean delivery*** | | | | | | | | | | |
|  | **Moderate Distribution in % of the cases removed** | **Moderate**  **Numbers**  **Total**  **N=169** | | **Severe**  **Distribution in % of the cases removed** | | | **Severe**  **Numbers**  **Total N=59** | | | |
| **Active first stage duration categorized** |  |  | |  | | |  | | | |
| <5.1 hours | 43.8% | 74 | | 52 % | | | 31 | | | |
| 5.1-7.5 hours | 17% | 29 | | 12.9% | | | 7 | | | |
| 7.5-10.1 | 17% | 29 | | 18.5% | | | 10 | | | |
| >10.1 hours | 21.9% | 37 | | 20% | | | 11 | | | |
